# Effects of Acute Low-Dose Exposure to the Chlorinated Flame Retardant Dechlorane 602 and Th1 and Th2 Immune Responses in Adult Male Mice

**DOI:** 10.1289/ehp.1510314

**Published:** 2016-04-15

**Authors:** Yu Feng, Jijing Tian, Heidi Qunhui Xie, Jianwen She, Sherry Li Xu, Tuan Xu, Wenjing Tian, Hualing Fu, Shuaizhang Li, Wuqun Tao, Lingyun Wang, Yangsheng Chen, Songyan Zhang, Wanglong Zhang, Tai L. Guo, Bin Zhao

**Affiliations:** 1 Research Center for Eco-Environmental Sciences, Chinese Academy of Sciences, Beijing, China; 2 University of Chinese Academy of Sciences, Beijing, China; 3 Environmental Health Laboratory Branch, California Department of Public Health, State of California, Richmond, California USA; 4 Department of Veterinary Biosciences and Diagnostic Imaging, College of Veterinary Medicine, University of Georgia, Athens, Georgia, USA

## Abstract

**Background:**

Although the chlorinated flame retardant Dechlorane (Dec) 602 has been detected in food, human blood, and breast milk, there is limited information on potential health effects, including possible immunotoxicity.

**Objectives:**

We determined the immunotoxic potential of Dec 602 in mice by examining the expression of phenotypic markers on thymocyte and splenic lymphocyte subsets, Th1/Th2 transcription factors, and the production of cytokines and antibodies.

**Methods:**

Adult male C57BL/6 mice were orally exposed to environmentally relevant doses of Dec 602 (1 and 10 μg/kg body weight per day) for 7 consecutive days. Thymocyte and splenic CD4 and CD8 subsets and splenocyte apoptosis were examined by flow cytometric analysis. Cytokine expression was measured at both the mRNA and the protein levels. Levels of the transcription factors Th1 (T-bet and STAT1) and Th2 (GATA3) were determined using quantitative real-time polymerase chain reaction (qPCR). Serum levels of immunoglobulins IgG1, IgG2a, IgG2b and IgE were measured by enzyme-linked immunosorbent assay (ELISA).

**Results:**

Splenic CD4+ and CD8+ T cell subsets were decreased compared with vehicle controls, and apoptosis was significantly increased in splenic CD4+ T cells. Expression (mRNA and protein) of Th2 cytokines [interleukin (IL)-4, IL-10, and IL-13] increased, and that of Th1 cytokines [IL-2, interferon (IFN)-γ and tumor necrosis factor (TNF)-α] decreased. The Th2 transcriptional factor GATA3 increased, whereas the Th1 transcriptional factors T-bet and STAT1 decreased. As additional indicators of the Th2-Th1 imbalance, production of IgG1 was significantly increased, whereas IgG2a was reduced.

**Conclusions:**

To our knowledge, we are the first to report evidence of the effects of Dec 602 on immune function in mice, with findings indicating that Dec 602 exposure favored Th2 responses and reduced Th1 function.

**Citation:**

Feng Y, Tian J, Xie HQ, She J, Xu SL, Xu T, Tian W, Fu H, Li S, Tao W, Wang L, Chen Y, Zhang S, Zhang W, Guo TL, Zhao B. 2016. Effects of acute low-dose exposure to the chlorinated flame retardant dechlorane 602 and Th1 and Th2 immune responses in adult male mice. Environ Health Perspect 124:1406–1413; http://dx.doi.org/10.1289/ehp.1510314

## Introduction

Dechlorane, or Mirex, is a chlorinated flame retardant that was widely used before concerns about its environmental effects prompted its replacement with analogs such as Dechlorane plus (DP), Dechlorane (Dec) 602, Dec 603, and Dec 604 (Sverko et al. 2011). However, these chemicals have also been detected in the environment in recent years. The environmental occurrence of Dec 602, 603, and 604 was first reported in 2009, and it is anticipated that they are persistent and bioaccumulative in the environment because of their high degree of chlorination (Shen et al. 2010). Little information is available on the production of Dec 602. It is listed on Canada’s Nondomestic Substances List and in the European Chemical Substances Information System, both of which indicate that Dec 602 is currently in use (Shen et al. 2011). Dec 602 is used in fiberglass-reinforced nylon-6 at 18% (Chanda and Roy 2006).

Dec 602 has been reported on in various environmental media, possibly because it is more bioaccumulative than DP and other chlorinated flame retardants that are currently in use (Dec 603 and Dec 604). In fish, the Dec 602 concentration was ~15–80 times higher than than that of DP, and the bioconcentration factor (BCF) of Dec 602 is ~40 times higher than that of DP (Shen et al. 2010). A study conducted in northern China further confirmed that the biota-sediment accumulation factor (BSAF), a measure of the bioaccumulation potential of hydrophobic organic compounds in aquatic biota, was higher for Dec 602 than for DP (Jia et al. 2011). Several studies have suggested that Dec 602 could be a global environmental contaminant. As an example of its ability to accumulate in biota from a remote environment, Dec 602 was detected in all samples of arctic beluga, but other types of dechloranes were not detected (Shen et al. 2012). Dec 602 has also been detected in air and seawater (Xian et al. 2011). Detection of Dec 602 in the environment suggests that humans are at a constant risk of Dec 602 exposure. Other chlorinated flame retardants such as Mirex have been banned owing to evidence of adverse health effects, including endocrine, immune, developmental, and reproductive toxicity (de Cock and van de Bor 2014). However, to our knowledge, no toxicity studies of Dec 602 on animals have been reported. Therefore, there is an urgent need to test the toxicity of this commonly used product.

The immune system has been considered to be a sensitive target when exposed to environmental pollutants (Inadera 2006). Studies have shown that persistent organic pollutants (POPs) and polybrominated diphenyl ethers (PBDEs) can exert acute toxic effects on the immune system (Fernandez-Salguero et al. 1995; Leijs et al. 2009). It was hypothesized that Dec 602 could target acquired immune responses and cause immune dysregulation (e.g., Th1/Th2 imbalance) following a short-term exposure. Here, we studied the effects of Dec 602 on the immune system, particularly on T cells in mice that had been systematically exposed to Dec 602 at doses of 1 and 10 μg/kg body weight per day; the results provided new insights into understanding the potential adverse effects of dechloranes and other chlorinated flame retardants.

## Methods

### Animals

Male C57BL/6 mice (6 weeks old, 20 ± 1 g) were purchased from Vital River Laboratories (VRL) and housed in the Animal Research Center of Tsinghua University under specific pathogen-free conditions, at a controlled temperature of 24°C ± 2°C and humidity of 50% ± 10%, with a cycle of 12 hr light and 12 hr dark. Animals were provided pellet feed and water *ad libitum* and were randomly assigned into three groups. The mice in the two treated groups were administered Dec 602 (Toronto Research Chemicals Inc.) in olive oil (low: 1 μg/kg body weight per day; high: 10 μg/kg body weight per day) by gavage. Controls received olive oil only.

The exposure doses used in the present study were selected to represent environmental exposures and were based on the following information. It has been reported that Dec 602 concentration in sediments was 6 ng/g dry weight, and this concentration accumulated to 34 ng/g lipid in fish from the same area (Shen et al. 2010). Given the higher BCF and the greater bioavailability of Dec 602, it is reasonable to suggest that Dec 602 concentrations are amplified along the food web (Shen et al. 2010). The level of lipid in fish from the Great Lakes was as high as 9.0 g lipid/100 g fish; thus, the concentration of Dec 602 in fish was as high as 3 ng/g fish (Turyk et al. 2012). Based on the concentration in fish, environmentally relevant doses of 1 and 10 μg/kg body weight (or 1 and 10 ng/g body weight) were used in the current mouse study. After 7 days exposure, the body weights were measured, sera were collected immediately after euthanasia, and thymus and spleen tissues were weighed and processed for further study. All animal protocols were approved by the Animal Care and Use Committee of Tsinghua University and were performed humanely for alleviation of suffering.

### Histopathological Examinations

Organs were collected from the mice and were fixed in 2.5% (v/v) glutaraldehyde-polyoxymethylene solution immediately after euthanasia. The tissue samples were dehydrated and embedded in paraffin wax. Serial paraffin sections (4 μm) were obtained and held at 37°C for more than 12 hr. The sections were immersed in three consecutive 5-min xylene washes to remove paraffin and were subsequently hydrated with five consecutive ethanol washes in descending order of concentration: 100%, 95%, 80%, 70%, and deionized water. The paraffin sections were then stained with hematoxylin-eosin (H&E), and changes in organizational structure were visualized using a light microscope with Leica Application Suite software (Leica Microsystems). Five sections were examined from each animal, and five animals were evaluated from each group.

### Flow Cytometry

Spleen and thymus were harvested, and single-cell suspensions were prepared by homogenization on 70-μm cell strainers (BD Falcon) using phosphate-buffered saline (PBS) with 2% fetal bovine serum (FBS). Spleen red blood cells were removed at 37^o^C by adding 2 mL of 1× lysing buffer (BD Biosciences) for 3 min. Cells were washed twice with cold PBS and stained for 30 min in an appropriately diluted antibody-staining solution. The antibodies used were rat anti-mouse CD3-PerCP-Cy^TM^5.5, rat anti-mouse CD4-FITC, and rat anti-mouse CD8-PE-Cy^TM^7; isotype controls were PerCP-Cy^TM^5.5 Rat IgG_2a_, FITC Rat IgG_2a_ and PE-Cy^TM^7 Rat IgG_2a_, respectively. For each 10^6^ cells, 0.5 μg antibody was used for staining. The antibodies were purchased from BD (BD Biosciences).

PE-Annexin V and 7-amino-actinomycin D (7-ADD) were used for the apoptosis analysis. Annexins are a family of intracellular proteins that bind to phosphatidylserine (PS). PS translocates to the outer membrane leaflet upon initiation of apoptosis. Necrotic or dead cells with increased membrane permeability are stained with 7-ADD. Thus, Annexin V single positive represents the early apoptotic stage of cells, and Annexin V/7-ADD double positive indicates the late stage of apoptosis. Cells were resuspended in 1× binding buffer and stained with PE-Annexin V and 7-ADD for 15 min at 25°C. Samples with a final volume of 400 μL were tested within 1 hr, and a total of 5,000 events were collected. CD4^+^ and CD8^+^ cells were first gated, and apoptotic cells were analyzed within the CD4^+^ and CD8^+^ cell subsets. Data were collected on a BD LSRII flow cytometer (BD Biosciences) and analyzed using Cell Quest software.

### RNA Isolation and Quantitative Real-Time Polymerase Chain Reaction

Total RNA was purified from spleen tissue using TRIzol reagent (Invitrogen), and 2 μg of RNA was reverse-transcribed using RevertAid First Strand cDNA Synthesis Kit (Thermo Scientific). Quantitative real-time polymerase chain reaction (qPCR) of interleukin (IL)-4, IL-10, IL-13, interferon (IFN)-γ, tumor necrosis factor (TNF)-α, IL-2, T-bet, STAT1, and GATA3 was performed on equal amounts of cDNA using GoTaq qPCR Master Mix (Promega). The housekeeping gene hypoxanthine-guanine phosphoribosyltransferase (HPRT) was used as an internal standard. The SYBR green signal was detected using a LightCycler®480 Instrument (Roche Diagnostics). The relative transcript expression levels were quantified using the ΔΔC_T_ method (Winer et al. 1999). The specificity of amplification was confirmed by melting curves and by gel electrophoresis.

### Luminex^®^ Assay

The concentrations of selected cytokines (IL-4, IL-10, IL-13, IFN-γ, TNF-α, and IL-2) in mouse sera and in spleen protein extracts obtained using NP40 cell lysis buffer (Life Technologies) were determined by Luminex® assay with a custom kit, the MCYTOMAG-70K Bead Panel (EMD Millipore Corporation). The instrument used was Luminex® 200^TM^ analyzer (Luminex), and data were collected using the MagPlex® program. Data were analyzed using Milliplex® Analyst 5.1 Software (Merck Millipore). All samples were tested in duplicate for the Luminex® assay.

### Enzyme-Linked Immunosorbent Assay

Levels of serum IgG_1_, IgG_2a_, IgG_2b_, and IgE were measured using enzyme-linked immunosorbent assay (ELISA) kits (eBioscience). Briefly, capture antibody (1:1,000 v/v) was coated separately on microtiter plates and incubated overnight at 4°C. After washing with PBS containing 0.05% TWEEN® 20 (PBST), microplate wells were blocked with 5% BSA at 20–25°C for 2 hr. After washing with PBST, diluted samples were added to the microplate and incubated for 2 hr at 25°C. After washing with PBST, horseradish peroxidase (HRP)-conjugated detection antibody was added to each well and incubated for 1 hr at 20–25°C. After washing with PBST, 3,3′,5,5′-tetramethylbenzidene (TMB) was added and incubated for 15 min at 20–25°C. The enzyme reaction was stopped with 1 M H_3_PO_4_. The optical density was read using a microplate fluorescence reader (Tecan Infinite F200 Pro) at a wavelength of 450 nm.

### Statistical Analysis

Experimental data were analyzed by one-way analysis of variance (ANOVA) or Dunnett’s test using GraphPad Prism 5 (GraphPad Software). The results are presented as the mean ± SD. Differences were classified as follows: *, *p* < 0.05 compared with the control; **, *p* < 0.01 compared with the control; and ***, *p* < 0.001 compared with the control.

## Results

### Body Weights, Relative Organ Weights, and Thymocyte Subsets Following Dec 602 Exposure

**Figure 1:**
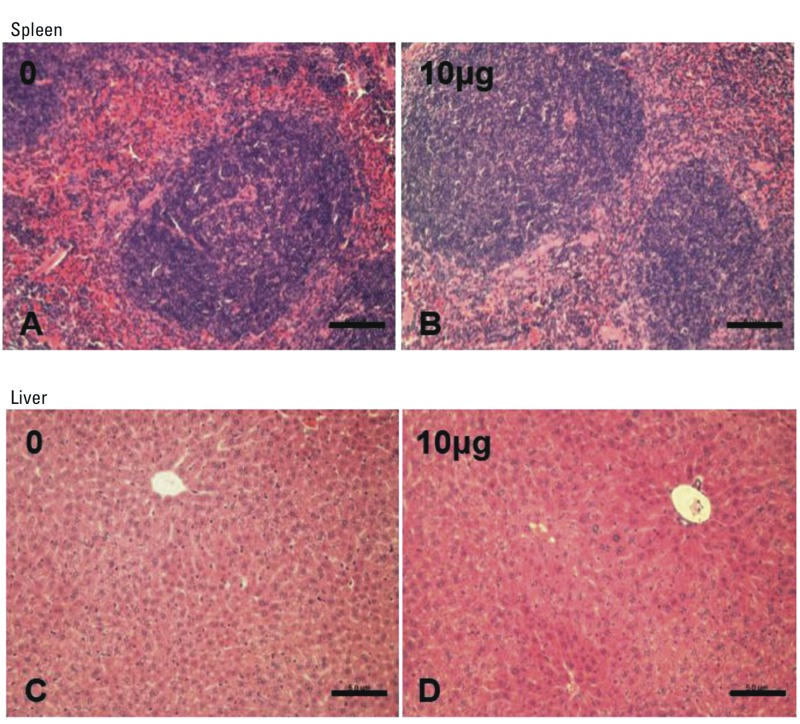
(*A*), (*B*), histopathological studies of spleen; (*C*), (*D*), histopathological studies of liver. Sections were stained with hematoxylin and eosin, and the images shown in the figure are representatives of all of the sections examined. Scale bar = 50 μm; 1 μg and 10 μg represent 1 μg/kg body weight and 10 μg/kg body weight, respectively.

**Figure 2:**
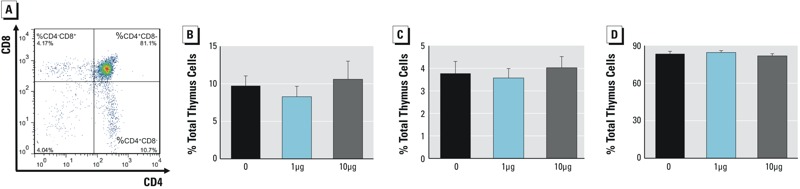
Effects of Dec 602 thymocyte differentiation. CD4^+^ and CD8^+^ T lymphocytes were tested using fluorescence activated cell sorting as described. (*A*) Representative dot plot of CD4 versus CD8; (*B*) %SP CD4^+^ thymocytes; (*C*) %SP CD8^+^ thymocytes; (*D*) %DP CD4^+^CD8^+^ thymocytes. *n* = 5–8; 1 μg and 10 μg represent 1 μg/kg body weight and 10 μg/kg body weight, respectively. Values shown in bar graphs are the means ± SD.

Exposure to Dec 602 had no significant effects on body weight, organ weights, and relative organ weights of brain, liver, thymus, spleen, and kidneys (Table 1). In addition, histopathological examination was conducted, and the results obtained from H&E staining showed no notable changes in spleen and liver (Figure 1A–D). To investigate the effects of Dec 602 on T cell differentiation, thymocytes were collected after 7 days of exposure. Immature double positive (DP) CD4^+^CD8^+^, mature single positive (SP) CD4^+^ and SP CD8^+^ thymocyte subsets were tested by fluoresence-activated cell sorting (FACS). Thymocyte subsets showed no significant changes after Dec 602 exposure (Figure 2A–D).

**Table 1:** Body weight, organ weights, and organ index following Dec 602 exposure (*n* = 5–8).

Parameters	0	1 μg/kg BW	10 μg/kg BW
BW (g)	18.83 ± 1.31	18.96 ± 1.33	18.41 ± 2.11
Brain (g)	0.41 ± 0.02	0.40 ± 0.02	0.41 ± 0.03
Liver (g)	1.04 ± 0.18	1.04 ± 0.14	0.98 ± 0.12
Thymus (g)	0.06 ± 0.01	0.06 ± 0.01	0.06 ± 0.01
Spleen (g)	0.06 ± 0.01	0.06 ± 0.01	0.06 ± 0.01
Kidneys (g)	0.27 ± 0.02	0.27 ± 0.02	0.27 ± 0.02
Brain (%BW)	2.17 ± 0.15	2.12 ± 0.17	2.27 ± 0.45
Liver (%BW)	5.48 ± 0.69	5.50 ± 0.52	5.44 ± 1.44
Thymus (%BW)	0.36 ± 0.10	0.35 ± 0.07	0.41 ± 0.20
Spleen (%BW)	0.32 ± 0.05	0.30 ± 0.04	0.33 ± 0.06
Kidney (%BW)	1.46 ± 0.07	1.41 ± 0.07	1.45 ± 0.25
BW, body weight.			

### Differential Splenic T Cell Subtypes

**Figure 3:**
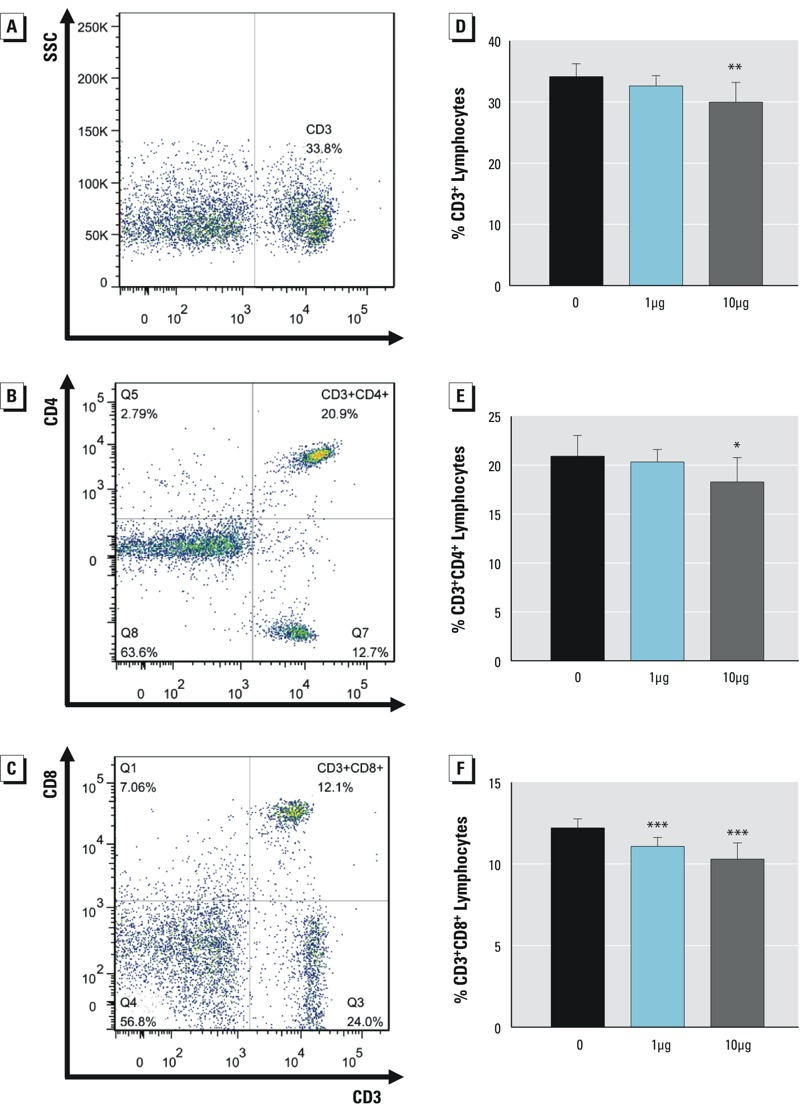
Dec 602 affected T cell development in spleen. CD3^+^, CD4^+^ and CD8^+^ T lymphocytes were tested using fluorescence activated cell sorting as described. (*A*), (*B*), and (*C*) Representative dot plots of CD3, CD4, and CD8, respectively; (*D*) %CD3^+^ lymphocytes; (*E*) %CD4^+^ lymphocytes; (*F*) %CD8^+^ lymphocytes. SSC, side-scattered light. *p < 0.05, **p < 0.01, ***p < 0.001; *n* = 5–8; 1 μg and 10 μg represent 1 μg/kg body weight and 10 μg/kg body weight, respectively. Values shown in bar graphs are the means ± SD.

To further test the effects of Dec 602 on the overall immune status, splenic T cell differentials were examined using FACS. When compared with the control group, CD3^+^ T cells were significantly lowered in the 10-μg/kg body weight treatment group (Figure 3A,D). No significant change in CD3^+^ cells was found in the 1-μg/kg body weight group. Subtypes of T cells were also tested. Similar changes occurred in the CD3^+^CD4^+^ T cell population, which was significantly decreased in the 10-μg/kg body weight group (Figure 3B,E; *p* < 0.05). CD3^+^CD8^+^ lymphocytes were significantly reduced (Figure 3C,F; *p* < 0.001) in both Dec 602-treated groups, indicating a possible impairment of cytotoxic T lymphocyte function.

### Spleen T cell Apoptosis

**Figure 4:**
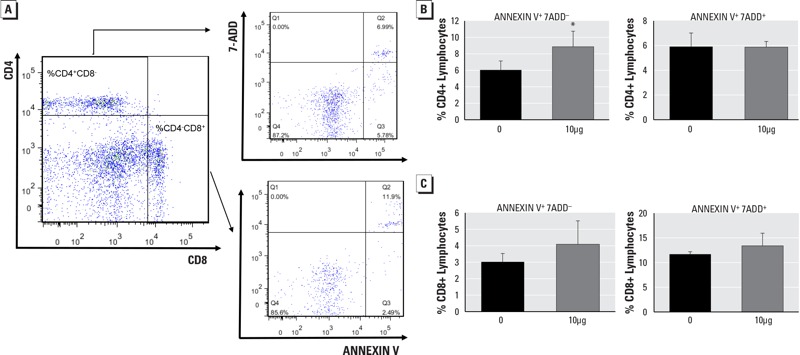
Dec 602 affected splenic T lymphocyte apoptosis. (*A*) CD4^+^ and CD8^+^ lymphocytes were gated and cell apoptosis was analyzed within CD4^+^ and CD8^+^ lymphocytes. Numbers in upper right and lower right quadrants represent end-stage (Annexin V^+^ and 7-ADD^+^) and early-stage apoptosis (Annexin V^+^ and 7-ADD^–^), respectively; (*B*) early- and end-stage apoptosis in CD4^+^ T lymphocyte subset; (*C*) early- and end-stage apoptosis in CD8^+^ cell subsets. *p < 0.05; *n* = 5–8; 1 μg and 10 μg represent 1 μg/kg body weight and 10 μg/kg body weight, respectively. Values shown in bar graphs are the means ± SD.

The above-mentioned results suggested that both CD4^+^ and CD8^+^ T cell subsets were decreased significantly in spleen following Dec602 exposure. To examine whether apoptosis was induced by Dec 602 in the 10-μg/kg body weight group, splenocytes were stained with the apoptosis markers Annexin V and 7-ADD and sorted by FACS (Figure 4A). The results showed that early-apoptotic CD4^+^ T cells (Annexin V^+^ and 7-ADD^–^) were significantly increased (*p* < 0.05) after Dec602 exposure (Figure 4B, left panel), but no difference was found in late-apoptotic cells (Annexin V^+^ and 7-ADD^+^; Figure 4B, right panel). We also observed nonsignificant (*p* > 0.05) increases in early- and late-apoptotic CD8^+^ T cells (increases of 1.25 ± 1.42% and 2.18 ± 2.56%, respectively) (Figure 4C).

### Th2 and Th1 Cytokine Expression

**Figure 5:**
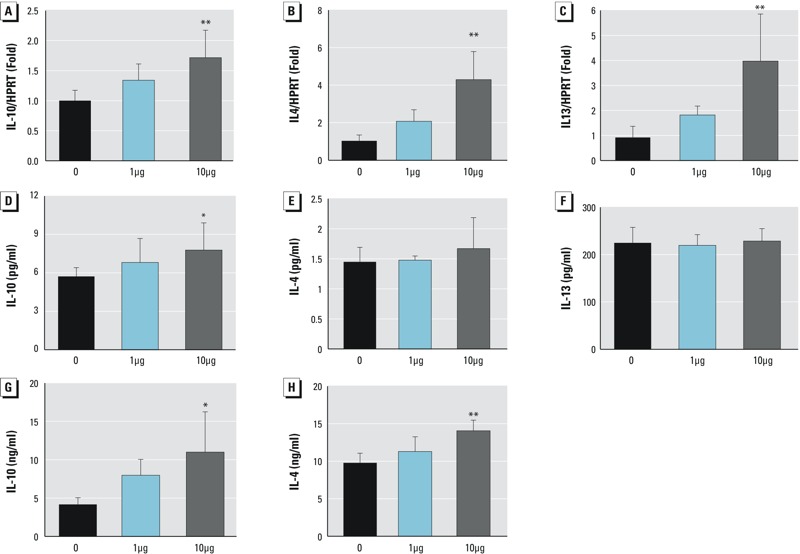
mRNA expression of Th2 cytokines in spleen and their protein levels in sera and spleen. Cytokines were examined at the mRNA level using quantitative real-time polymerase chain reaction, and they were examined at the protein level in sera and spleen using Luminex^®^ assays as described. (*A*), (*B*), (*C*) mRNA expression of interleukin (IL)-10, IL-4 and IL-13, respectively; (*D*), (*E*), (*F*), protein levels of IL-10, IL-4 and IL-13 in sera, respectively; (*G*), (*H*) protein levels of IL-10 and IL-4 in spleen extracts, respectively. HPRT, hypoxanthine phosphoribosyltransferase 1; *p < 0.05, **p < 0.01; *n* = 5–8; 1 μg and 10 μg represent 1 μg/kg body weight and 10 μg/kg body weight, respectively. Values shown in bar graphs are the means ± SD.

After Dec 602 exposure, both Th1 and Th2 cytokines were tested at the mRNA level in spleen (qPCR) and at the protein level in sera and in spleen protein extracts (Luminex® assay). Typical Th2 cytokines including IL-10, IL-13, and IL-4 were examined, and significant increases were found for all of these cytokines at the mRNA level (Figure 5A–C). IL-10 mRNA (Figure 5A) was 1.73 ± 0.45 times higher (*p* < 0.01) in the 10-μg/kg body weight group. IL-4 (Figure 5B) and IL-13 (Figure 5C) mRNA expression was also increased significantly. In the 1-μg/kg body weight treatment group, we observed nonsignificant increases in IL-4 (2.07 ± 0.62 times) (Figure 5B) and IL-13 (1.85 ± 0.36 times) (Figure 5C), and in the 10-μg/kg body weight group, IL-4 and IL-13 expression increased by 4.29 ± 1.49 times (*p* < 0.01) and 3.98 ± 1.88 times (*p* < 0.05), respectively. The IL-10 protein level in sera increased significantly in the 10-μg/kg body weight treatment group (Figure 5D; *p* < 0.05), whereas IL-4 and IL-13 levels were not significantly different from those in controls (Figure 5E,F). Both IL-10 and IL-4 levels in spleen protein extracts increased significantly in the 10-μg/kg body weight treatment group (Figure 5G; *p* < 0.05; Figure 5H; *p* < 0.01, respectively).

**Figure 6:**
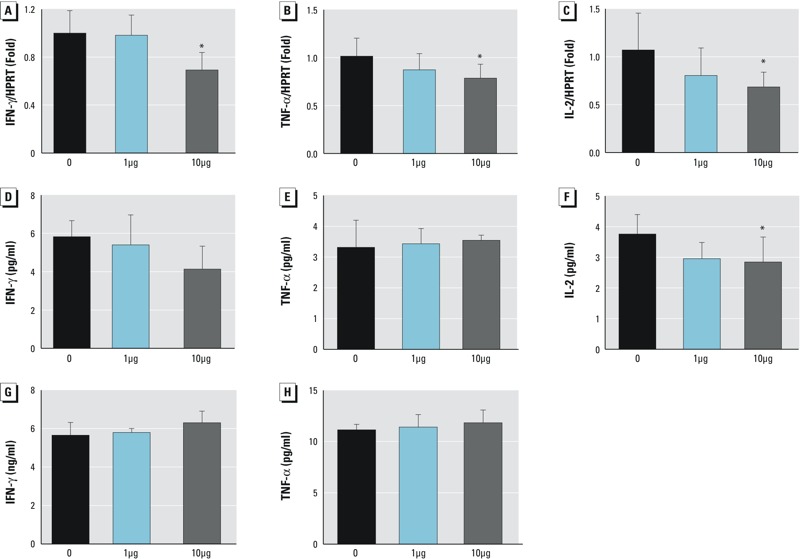
mRNA expression of Th1 cytokines in spleen and their protein levels in sera and spleen. Cytokines were examined at the mRNA level using quantitative real-time polymerase chain reaction and at the protein level in sera and spleen using Luminex^®^ assays as described. (*A*), (*B*), (*C*) mRNA expression of interferon (IFN)-γ, tumor necrosis factor (TNF)-α and interleukin (IL)-2, respectively; (*D*), (*E*), (*F*), protein levels of IFN-γ, TNF-α and IL-2 in sera, respectively; (*G*), (*H*), protein levels of IFN-γ and TNF-α in spleen extracts, respectively. HPRT, hypoxanthine phosphoribosyltransferase 1; *p < 0.05; *n* = 5–8; 1 μg and 10 μg represent 1 μg/kg body weight and 10 μg/kg body weight, respectively. Values shown in bar graphs are the means ± SD.

With regard to Th1 cytokines, the mRNA levels of IFN-γ (Figure 6A), TNF-α (Figure 6B), and IL-2 (Figure 6C) decreased significantly in the 10-μg/kg body weight treatment group (*p* < 0.05). No obvious decreases were observed in the 1-μg/kg body weight treatment group. Luminex® analysis of sera showed a significant decrease of IL-2 at the protein level in the 10-μg/kg body weight treatment group (Figure 6F; *p* < 0.05). However, IFN-γ and TNF-α levels in both sera and spleen protein extracts showed no significant differences from controls in either treatment group (Figure 6D,E,G,H).

### Th2 and Th1 Transcription Factors

**Figure 7:**
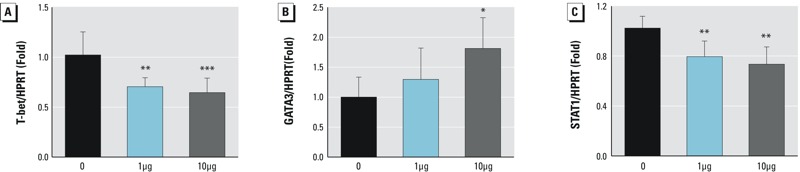
Effects of Dec 602 on Th1/Th2 transcriptional factor expression. T-bet [Th1; (*A*)], GATA3 [Th2; (*B*)] and the key Th1 regulator STAT1 (*C*) were tested at the mRNA level using quantitative real-time polymerase chain reaction as described. HPRT, hypoxanthine phosphoribosyltransferase 1; *p < 0.05,****p < 0.01,*****p < 0.001; *n* = 3–5; 1 μg and 10 μg represent 1 μg/kg body weight and 10 μg/kg body weight, respectively. Values shown in bar graphs are the means ± SD.

Patterns of cytokine expression suggested a shift in the Th1/Th2 balance after Dec 602 exposure. Therefore, we examined the expression of transcriptional factors for Th1 (T-bet, STAT1) and Th2 (GATA3) cells in the spleen using qPCR. We found that T-bet and STAT1 decreased significantly in both treatment groups (Figure 7A,C; *p* < 0.01 and *p* < 0.001, respectively), whereas GATA3 increased significantly in the 10-μg/kg body weight group (Figure 7B; *p* < 0.05).

### Serum IgG_1_, IgG_2a_, IgG_2b_, and IgE Levels

**Figure 8:**
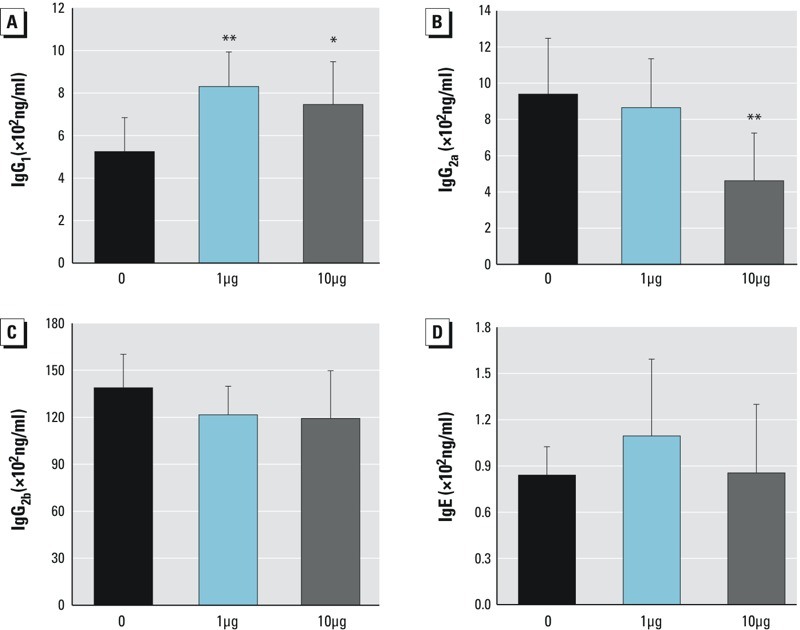
Effects of Dec 602 on serum levels of immunoglobulin G (IgG) subclasses and IgE. Enzyme-linked immunosorbent assays were performed as described. (*A*) IgG_1_, (*B*) IgG_2a_, (*C*) IgG_2b_, (*D*) IgE. *p < 0.05, **p < 0.01; *n* = 5–8; 1 μg and 10 μg represent 1 μg/kg body weight and 10 μg/kg body weight, respectively. Values shown in bar graphs are the means ± SD.

Th1 cells induce production of IgG_2a_ antibody, and Th2 cells promote IgE and IgG_1_ (Peng et al. 2002). IgG_2b_ can be produced in response to either Th1 or Th2 under different conditions (Bennett 2003; Dixit et al. 2014). Thus, serum antibody levels of IgG_1_, IgG_2a_, IgG_2b_, and IgE were tested using ELISA. The level of IgG_1_ (Figure 8A) increased by 58 ± 2% in the 1-μg/kg body weight group (*p* < 0.01) and by 47 ± 2% in the 10-μg/kg body weight group (*p* < 0.05). IgG_2a_ (Figure 8B) showed a significant decrease of 51 ± 3% in the 10-μg/kg body weight group (*p* < 0.01). There were no significant differences in IgE or IgG_2b_ production for either treatment group relative to controls (Figure 8C,D, respectively).

## Discussion

Although dechloranes have been introduced into the environment for decades, there is limited information about their potential toxicity. Previous studies regarding DP toxicity used relatively high doses, for example, 5 × 10^5^, 2 × 10^6^, and 5 × 10^6^ μg/kg body weight for 10 days (Wu et al. 2012) and 10^3^, 10^4^ and 10^5^ μg/kg body weight for 90 days (Li et al. 2013), and these studies showed undetectable or low toxicity of DP. However, considering their daily usage, low-dose effects of DP as well as those of other isomers should be investigated (Zhu et al. 2007). After 7 consecutive days of relatively low-dose Dec 602 exposure, our studies showed decreased percentages of both CD4^+^ and CD8^+^ T cell subsets in spleen. Several reasons could have led to this changing profile of T cells, and here, we showed that splenic T cell apoptosis might be one of the underlying mechanisms.

T cells regulate immune responses by secreting cytokines. IFN-γ secretion by Th1 cells inhibits Th2 cell proliferation, and IL-4 secretion by Th2 cells can inhibit Th1 proliferation. In addition, the cytokine IL-10 modulates the Th1/Th2 balance in favor of Th2 cell responses by inhibiting the production of IFN-γ by Th1 lymphocytes (Asadullah et al. 2003). Dec 602 exposure at 10 μg/kg body weight caused increased IL-10 and IL-4 mRNA expression and decreased IFN-γ expression. This pattern of cytokine expression is consistent with a shift in favor of Th2 cell proliferation. Additional studies on the expression of transcriptional factors of both Th1 and Th2 also support the notion that Dec 602 caused Th1/Th2 dysregulation. The STAT1 pathway is one of the signal transduction pathways that controls T-bet expression (Afkarian et al. 2002). STAT1 mRNA expression was significantly decreased following Dec 602 exposure, suggesting the possible involvement of the IFN-γ/STAT1 pathway. IgG_1_ and IgG_2a_ production can reflect Th1/Th2 reactivity. Importantly, IgG_1_ increased while IgG_2a_ decreased, consistent with an increase in Th2 and a decrease in Th1 responses, respectively. Increased ratios of GATA3/T-bet and IgG_1_/IgG_2a_, together with the observed pattern of cytokine expression suggested that the Th1/Th2 balance was shifted to the Th2 phenotype, that is, that T cell differentiation may have been modulated by Dec 602. Dysregulation of the Th1/Th2 balance may alter cytokine patterns and lead to aberrant immune responses.

One of the reported mechanisms for tumor escape is that recruited immune cells express cytokines such as IL-13 and IL-4 to generate a tumor-favoring microenvironment. In addition, high concentrations of TNF-α have antitumor effects, whereas constant low-level expression of TNF-α can induce a tumor phenotype (van Horssen et al. 2006). IL-2 plays an important role in maintaining T cell activities, including anti-tumor activity. IL-2 administration is an effective cancer immunotherapy (Rosenberg 2014). Mitogen-activated protein kinase (MAPK) pathways have critical roles in the production of both IL-2 and TNF-α (Chi et al. 2006; Li et al. 1999). Dec 602 may negatively regulate MAPK activity, and further study should be conducted to verify the molecular mechanisms in Dec 602–induced dysregulation of both cytokines. In the present study, we observed increased IL-13, IL-4, and IL-10 levels and decreased IL-2, IFN-γ, and TNF-α after Dec 602 exposure, findings that support the need for additional research to determine whether exposure might promote a dysregulated immune environment that could increase vulnerability to tumor evasion (Vesely et al. 2011). This situation may be further exacerbated by decreased IFN-γ levels, as observed in mice exposed to Dec 602 (Zhang et al. 2008).

Dec 602 has been identified at various concentrations in dietary products such as meat and fish (Kim et al. 2014). Fifty percent of sera from Norwegian women contained measurable Dec 602 (Cequier et al. 2015). In another study, Dec 602 was detected in human sera and breast milk collected from approximately 100 Canadian women during 2007–2009 (the maximum value in sera was ≤ 5.7 ng/g lipid, and the median concentrations were 0.53 and 0.23 ng/g lipid in sera and breast milk, respectively), suggesting the potential for mother-to-fetus transmission (Zhou et al. 2014). In our study of adult male mice, short-term and low-dose exposure to Dec 602 appeared to alter the Th1/Th2 balance. However, responses to Dec 602 may differ between newborns and adults (as well as between humans and mice); therefore, further experiments are needed to evaluate differences in the effects of Dec 602 according to developmental stages.

Murine models have long been used to closely mimic human inflammatory conditions (Takao and Miyakawa 2015). If Dec 602 is a potent immunosuppressive chemical in mice, it is likely that it would be immunotoxic to humans as well. However, there are major differences in both the innate and cellular immune responses of mice and humans (Mestas and Hughes 2004), and future studies should also use human cells to investigate the immunotoxic effects of Dec 602. To our knowledge, no one has conducted experiments to measure background Dec 602 levels in the serum of experimental animals or serum levels of Dec 602 in animals after Dec 602 exposure. We are developing a credible and feasible method to determine the serum concentration of Dec 602 in mice, and it is our intention to further investigate the serum and tissue concentrations of Dec 602 in mice.

## Conclusion

In summary, our findings indicated that short-term Dec 602 exposure altered immune responses in adult male mice. Th1 suppression may result in attenuated host resistance that increases the body’s vulnerability to infections and cancer (Ilinskaya and Dobrovolskaia 2014). In addition, enhanced Th2 response can trigger airway hyperresponsiveness (Wills-Karp et al. 1998), resulting in a potential risk of allergic asthma. Given the immunotoxic effects of Dec 602, further studies using various animal models to determine cancer and asthma risks are warranted.
